# Editorial: Advances of imaging techniques in identifying malignancy in thyroid nodules

**DOI:** 10.3389/fendo.2023.1188250

**Published:** 2023-04-14

**Authors:** Hendra Zufry, Emerita Andres Barrenechea, Aisyah Elliyanti

**Affiliations:** ^1^ Division of Endocrinology, Metabolism, and Diabetes, Thyroid Center, Department of Internal Medicine, School of Medicine, Universitas Syiah Kuala/Dr. Zainoel Abidin General Teaching Hospital, Banda Aceh, Indonesia; ^2^ Innovation and Research Center of Endocrinology, School of Medicine, Universitas Syiah Kuala, Banda Aceh, Indonesia; ^3^ Department of Nuclear Medicine and Research, Veterans Memorial Medical Center, Quezon City, Philippines; ^4^ Department of Nuclear Medicine and PET, St. Luke's Medical Center, Quezon City, Philippines; ^5^ Nuclear Medicine Division of Radiology Department, Faculty of Medicine, Universitas Andalas/Dr. M. Djamil Hospital, Padang, Indonesia

**Keywords:** thyroid nodules, artificial intelligence, deep learning, advanced thyroid imaging, real-world evidence, TIRADS

The management of thyroid nodules (TNs) has gained attention due to its increasingly high incidence rate (1). It was estimated that over 586,000 people were diagnosed with TNs in 2020 (2). TNs have been identified more frequently in recent years due to increased sensitivity in imaging techniques (3). TNs patients are diagnosed by history taking and physical examination, ultrasonography, Thyroid -stimulating hormone (TSH) serum measurement, fine needle aspiration, and scintigraphy (4). Most TNs proved benign; however, 7 to 15% of TNs are malignant, causing mortality if not treated properly (5, 6). Hence, diagnostic tools are most necessary to differentiate benign and malignant TNs to provide optimal treatment.

We invited researchers worldwide to address advanced imaging techniques to differentiate TNs and innovations in thyroid imaging approaches for therapy. We received diverse and insightful nine manuscripts involving 68 authors from various backgrounds. The Research Topic was divided into three areas of research: 1) Artificial intelligence prediction models compared to real-world evidence; 2) Advanced ultrasonography from grey-scale to contrast-enhanced ultrasonography; and 3) Ultrasonography (US) characterization for TNs and lymph node metastasis. This research aimed to assess the malignancy risk, identify poor prognostic factors, and determine the optimal management plan for patients.

The US is an operator-dependent technique, and the imaging results’ interpretation is affected by the radiologists’ experience (5). To reduce a variation of US interpretations between operators, recent studies have investigated the role of a deep-learning prediction model to assess thyroid nodule malignancy risk and metastatic lymph nodes. Zhang et al. invented a deep learning prediction model for diagnosing papillary thyroid carcinoma (PTC) malignancy that incorporated many variables – demographic, serological, ultrasound, and biopsy data of 2,029 patients. The study reported an excellent predictive ability to differentiate between benign and malignant PTC. Furthermore, Chang et al. developed a deep learning model integrated with clinical and ultrasound factors to predict central lymph node metastasis (CLNM) in PTC by using the dataset of 3,359 CLNM patients, and the study may have superior clinical predictive tools compared to other deep learning-based models.

Even though the deep learning prediction model has promising predictive capabilities, the US remains the conservative diagnostic modality of TNs malignancy, especially in areas with limited healthcare facilities (5). Feng et al. explored the effectiveness of ultrasound grayscale ratio (UGSR) to differentiate benign and malignant TNs in patients with Hashimoto’s thyroiditis (HT). The UGSR showed an excellent diagnostic result to determine TNs malignancy in HT patients. Wang et al. studied the US to detect partially cystic thyroid nodules (PCTNs). The study described US characteristic features for malignant and benign PCTNs with reliable diagnostic ability. Chen et al. examined the role of contrast-enhanced ultrasonography (CEUS) to differentiate mummified TNs from other malignant TNs. However, a large-scale population with a multicenter prospective study is needed to verify the specificity and sensitivity of the CEUS findings.

The US features a high prediction tool for malignant TNs (1). Thyroid Imaging Reporting and Data System (TIRADS) provides US prediction tools for TNs malignancy – the most popular are ACR-TIRADS, EU-TIRADS, and K-TIRADS. A study by Chen et al. compared malignancy risk stratification of TNs for C-TIRADS, K-TIRADS, and ACR-TIRADS. Results showed that C-TIRADS has outstanding performance in the malignancy risk stratifications by optimized cut-off value compared with other TIRADS. Another interesting study was provided by Zhou et al. and Yao et al., where both studies support each other. Zhou et al. evaluated the sonographic characteristics and risk factors of CLNM in 2,376 patients. Although the US reported excellent sensitivity and specificity, Yao et al. suggested that a combination of US and CT-Scan showed better results in diagnosing CLNM rather than the US alone. Moreover, Liu et al. determined the optimal imaging time after Iodine ^-131^ treatment; imaging sensitivity on day three detected residual thyroid tissue and on day seven and day ten detected cervical lymph nodes and lung metastases.

These findings highlight how advanced imaging techniques provide a more expansive view of TNs diagnosis. Although the artificial intelligence prediction model had better accuracy than the conventional ones, it may be limited to developing countries. We encourage more accessibility with exact accuracy tools such as a web-based deep learning model, which will be clinically applicable in iodine-deficit regions – mainly in developing countries. Nevertheless, we are confident that all selected studies on this Research Topic bring essential and enduring contributions to differentiate thyroid nodule malignancy.

## Author contributions

HZ: conceptualizing, writing the original draft, writing–review, and editing. EB: writing–review, and editing. AE: writing–review, and editing. All authors approved the submitted version.
